# Adrenergic blockers, statins, and non-steroidal anti-inflammatory drugs are associated with later age at onset in Parkinson’s disease

**DOI:** 10.1007/s00415-025-12989-2

**Published:** 2025-03-06

**Authors:** Camille Malatt, Helia Maghzi, Elliot Hogg, Echo Tan, Ishani Khatiwala, Michele Tagliati

**Affiliations:** Department of Neurology, Cedars-Sinai Medical Center, 127 S. San Vicente Blvd. #A6600, Los Angeles, CA 90048 US

**Keywords:** Parkinson’s disease, Age at onset, Adrenergic blockers, Statins, Non-steroidal anti-inflammatory drugs (NSAIDs)

## Abstract

**Background:**

Several factors have been shown to modify the risk of developing Parkinson’s disease (PD), including commonly prescribed medications. However, there is little data describing their correlation with age at onset (AAO) of clinical symptoms. The objective of this study was to evaluate the association of treatment with anti-hypertensives, non-steroidal anti-inflammatories (NSAIDs), statins, as well as smoking and family history of PD with AAO in a large clinical cohort.

**Methods:**

A retrospective review of 1201 initial encounters collected information on known risk-modulating factors for PD, including smoking status and family history, anti-hypertensives, statins, NSAIDs, anti-diabetic medications, and beta-agonists. In addition to general exposure, we determined whether medications of interest were started before or after onset of symptoms. Mean AAO was calculated for each set of variables. T-test and multiple regression analyses were used to evaluate association with AAO.

**Results:**

Exposure to all studied medications showed a strong correlation with older PD AAO, except for smoking and family history, which correlated with younger AAO. Multiple regression analysis identified exposure to adrenergic blockers (AB) (β = 5.7), statins (β = 5.6), and NSAIDs (β = 4.1) as the strongest independent predictors of older PD AAO (p < 0.001). Patients who were started on AB prior to onset of PD symptoms showed the largest average delay of PD AAO (at 72.3 ± 10.1 years), almost 10 years later as compared with those not on AB (62.7 ± 10.7 years) or those who started taking AB after onset of symptoms (63.0 ± 10.6 years).

**Conclusions:**

Multiple common medications are associated with a considerable delay of PD onset.

**Supplementary Information:**

The online version contains supplementary material available at 10.1007/s00415-025-12989-2.

## Introduction

The risk of developing Parkinson’s disease (PD), a progressive neurodegenerative disorder characterized by bradykinesia, muscle rigidity, and resting tremor, is age dependent, with increasing incidence until age 80, after which it tends to decline. While the pathogenesis of PD is still unclear, genetic factors, cumulative exposure to environmental factors, or age-dependent biological factors may affect its development. Several risk and protective factors have been associated with PD, but few have been related specifically to age at onset (AAO). There is substantial evidence that monogenic forms of PD frequently determine early disease AAO [1]. Caffeine intake and smoking have been instead associated with delayed PD AAO [2].

Several common medications, including statins [3], adrenergic modulating drugs [4], renin-angiotensin system inhibitors [5], calcium channel blockers (CCBs) [6], and non-steroidal anti-inflammatories (NSAIDs)[7] have been associated with a reduced PD risk. Similarly, it has been proposed that beta-agonists may reduce the risk of PD, while beta-blockers (particularly propranolol) may increase it [8], although this association has been debated [4].

Despite their putative role in modulating PD risk, little data has described the relationship between use of these medications and PD AAO. Aspirin was associated with a 5-year delay in AAO in one study [2], and patients with type-2 diabetes (T2D) on anti-diabetic treatment developed PD about 7 years later than patients who did not have T2D in another investigation [9]. Statins have also been shown to delay AAO by 9 years [10]. Given the current lack of disease-modifying therapies for PD, any medication that may delay the onset of PD, and therefore prevent years of disability, should be further studied. In addition, the mechanism of action of such medications may shed light into the pathogenesis of PD. In a large PD cohort, we investigated the association of AAO with several classes of anti-hypertensives, adrenergic agonists, statins, NSAIDs and anti-diabetics, as well as smoking and family history of PD.

## Methods

### Participants and study design

We retrospectively reviewed medical records of PD patients examined by movement specialists at Cedars-Sinai Medical Center from October 2010 to December 2021. At first encounter, a standardized template recorded information about time of onset of PD symptoms (defined as the time when the first cardinal motor symptoms appeared), current medications, motor and non-motor symptoms. This research was approved by the local institutional review board.

### Risk-modulating factors

The template collected information on known risk-modulating factors for PD, including smoking status, family history (FH), anti-hypertensives – specifically adrenergic blockers (AB), angiotensin-converting-enzyme inhibitors (ACE-Is), angiotensin II receptor blockers (ARBs), CCBs, and diuretics, beta-agonists, statins, NSAIDs and anti-diabetic medications. A smoking history within 10 years preceding PD onset was classified as a positive smoking history, while a smoking history prior to this was considered equivalent to no smoking history. Of note, our center did not start screening for genetic variants until late 2020. Given the very low prevalence of monogenic PD variants in large studies[1], we believe that lack of genetic screening did not alter the integrity of our data. In addition to exposure at time of clinical encounter, we determined whether medications of interest were started before or after onset of PD symptoms by review of medical records. In the absence of documented dates, we obtained information on medication starting dates by interviewing patients. Medications prescribed “as needed” were not included as an exposure.

### Statistical analysis

Parametric T-tests were performed to compare the means of AAO between different groups. A Shapiro–Wilk test was used to confirm normality of data distribution. Reported p values are descriptive as they are not corrected for multiple testing, and results are exploratory. Because multiple risk-modulating factors could coexist in each subject, a multiple regression analysis was used to evaluate relative influence. We applied a model using AAO as dependent variable, with gender and risk-modulating factors as binary covariates (R Core Team). Subjects that had missing data values regarding specific medication start dates were not included in the regression analysis.

## Results

We recorded 1201 consecutive initial encounters resulting in a diagnosis of PD. 763 patients (63.5%) were male. Average age at initial visit was 69.8 years (SD = 10.2, Range = 35–95), while the mean PD AAO was 63.7 years (SD = 10.9, Range = 26–93). Comorbidities included hypertension in 502 patients (41.7%), hyperlipidemia in 406 patients (33.8%), coronary artery disease in 154 patients (12.8%), atrial fibrillation in 87 patients (7.2%), myocardial infarction in 39 patients (3.2%), and congestive heart failure in 28 patients (2.3%). Results are presented in order of relative frequency of exposure to the various PD risk-modulating factors (Table 1).

**Table 1:** Mean PD age at onset (AAO) based on exposure status to studied variables

Variable	Mean AAO				Beta*	p value*
	Subjects exposed prior to PD onset (SD)	N	Subjects not exposed prior of PD onset (SD)	N		
Statins	70.8 (8.3)	178	61.7 (11.1)	839	5.62	4.8 × 10^–5^
NSAIDs	70.6 (8.2)	146	62.3 (11.1)	906	4.06	8.1 × 10^–4^
ACE-Is/ARBs	69.5 (8.7)	119	62.8 (11.0)	935	1.59	0.20
Adrenergic Blockers	72.3 (8.7)	99	62.8 (11.0)	1035	5.71	5.2 × 10^–7^
Diuretics	70.5 (9.6)	45	63.6 (10.9)	1079	0.87	0.64
Calcium Channel Blockers	71.5 (9.9)	46	62.8 (10.9)	1097	2.84	0.11
Anti-diabetics	68.5 (9.8)	48	63.7 (10.9)	1099	−1.80	0.34
Beta-agonists	68.8 (9.8)	25	63.6 (10.9)	1162	0.77	0.73
Smoking History	59.2 (8.2)	56	64.0 (10.8)	1085	−5.96	0.001
Family History	61.8 (10.2)	310	64.4 (11.1)	835	−1.56	0.074

*PD* Parkinson’s disease, *AAO* Age at onset, *NSAIDs* Non-steroidal anti-inflammatories, *ACE-Is/ARBs* ACE-inhibitors/angiotensin ii receptor blockers

^*^Beta and p values from the multiple regression analysis

### Statins

**Fig. 1:**
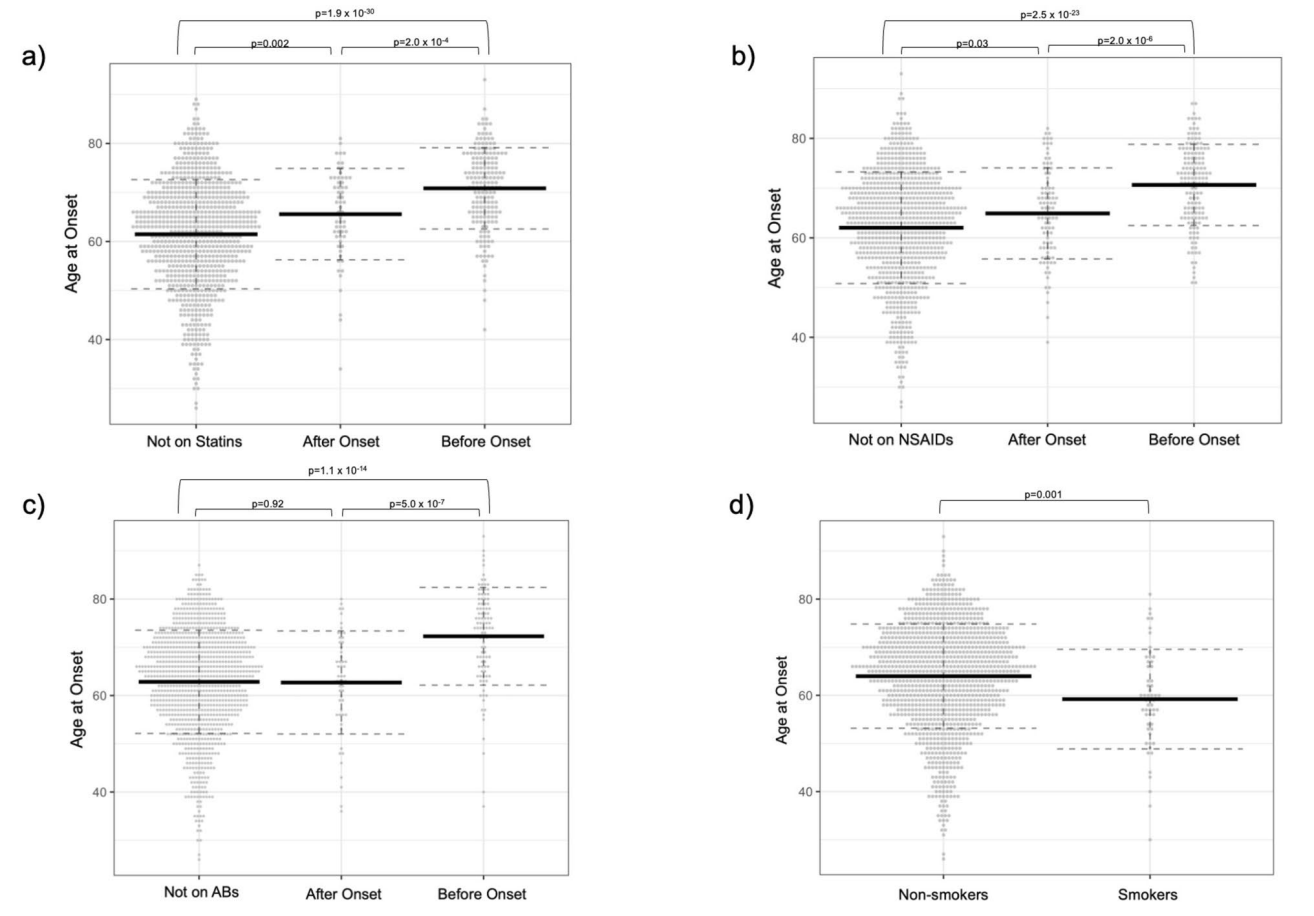
Dot plots showing the association of PD AAO with a) statin intake, b) NSAID intake, c) adrenergic blockers intake, and d) smoking history, which were the four strongest independent predictors of AAO in the multiple regression analysis. Black bar is the mean AAO and dotted bars are ± 1 SD. Exposure to these medications was associated with a delay in AAO when compared to those who were not on the medication or were started after developing PD symptoms, while smoking history was associated with an earlier AAO when compared to those without a recent smoking history

At time of first encounter, 422 patients were taking statins. Of these, 178 were on statins prior to the onset of PD motor symptoms and 60 were started after. A start date could not be ascertained for 184 patients. Those who were on statins prior to onset of PD symptoms had later AAO (mean AAO = 70.8 ± 8.3 years) compared to those who were not on statins (mean AAO = 61.5 ± 11.2 years; p = 1.9 × 10^–30^). Those who started taking statins after onset of PD symptoms had later AAO (mean 65.3 ± 9.4 years) than those never exposed (p = 0.002), but earlier than those on statins prior to PD symptom onset (p = 0.0002; Fig. 1a).

### Nonsteroidal anti-inflammatory drugs (NSAIDs)

333 patients were taking NSAIDs at time of first clinical encounter. Of these, 146 were on NSAIDs prior to the onset of PD motor symptoms and 93 were started after. Details regarding when NSAIDs were started could not be ascertained for 148 patients. Types of NSAIDs included aspirin, ibuprofen, meloxicam, naproxen, celecoxib, and ketoprofen, although the majority of patients were on aspirin alone. PD patients who were on NSAIDs prior to onset of PD symptoms had later AAO (mean AAO = 70.6 ± 8.2 years) as compared to those who were not on NSAIDs (mean AAO = 62.0 ± 11.2 years; p = 2.5 × 10^–23^). Those who started taking NSAIDs after onset of PD symptoms had a later AAO (mean AAO = 64.7 ± 9.3) than those never exposed (p = 0.006), but earlier than those on NSAIDs prior to onset of PD symptoms (p = 0.000002; Fig. 1b).

### ACE-inhibitors/angiotensin II receptor blockers

Based on the common mechanism of action, we grouped patients taking ACE-I and ARBs medications. 306 patients were taking ACE-I/ARBs at time of initial encounter. Of these, 119 patients were on ACE-I/ARBs prior to the onset of PD motor symptoms and 40 were started after. Details regarding when ACE-I/ARBs were started could not be ascertained for 147 patients. Patients with PD who were on ACE-Is/ARBs prior to onset of PD symptoms had later AAO (mean AAO = 69.5 ± 8.7 years) compared to those who were not on ACE-I/ARBs (mean AAO = 62.6 ± 11.1 years; p = 3.4 × 10^–13^). Those who started taking ACE-Is/ARBs after onset of PD symptoms had a later AAO (mean AAO = 66.7 ± 8.4 years) than those never exposed (p = 0.003) and were younger at onset than those on ACE-Is/ARBs prior to onset of PD symptoms (p = 0.1).

### Adrenergic blockers

At time of first encounter, 219 patients were taking AB (plus 35 taking propranolol, which were analyzed separately). Of these, 99 patients were on AB prior to the onset of PD motor symptoms and 53 were started after. Details regarding AB start could not be ascertained for 67 patients. The types of AB included atenolol (12.5 mg-50 mg daily), bisoprolol (2.5–5 mg daily), carvedilol (3.125 mg-25 mg daily), labetalol (200–600 mg daily), metoprolol succinate (12.5–100 mg daily), metoprolol tartrate (25–100 mg daily), nebivolol (2.5–20 mg daily), and sotalol (160–240 mg daily). A precise duration of treatment could not be ascertained with sufficient detail, as well as titration or tapering schedules. Patients with PD who were on AB prior to onset of PD symptoms had later AAO (mean AAO = 72.3 ± 10.1 years) compared to those who were not on AB (mean AAO = 62.7 ± 10.7 years; p = 1.1 × 10^–14^). Those who started taking AB after onset of PD symptoms had a mean AAO similar to those never exposed to AB (mean AAO = 63.0 ± 10.6 years; p = 0.92; Fig. 1c).

### Propranolol

35 patients were taking propranolol at time of first encounter. Of these, 31 had propranolol prescribed after onset of tremor that turned out to be part of their PD syndrome. One patient was prescribed propranolol prior to onset of PD symptoms as they had been diagnosed with essential tremor 50 years prior. Of the remaining 3 patients whose start times were unclear, one patient had documentation that propranolol was started for hypertension and two patients had diagnoses of hypertension in the chart, but we were unable to clarify the reason for propranolol treatment. Patients on propranolol had a mean AAO of 63.4 ± 11.3 years, indistinguishable from the population of PD patients never exposed to AB (p = 0.71).

### Diuretics

154 patients were taking diuretics at time of first encounter. Of these, 45 were on diuretics prior to the onset of PD symptoms and 32 were started after. Details regarding when diuretics were started could not be ascertained for 77 patients. Patients with PD who were on diuretics prior to onset of PD symptoms had later AAO (mean AAO = 70.5 ± 9.6 years) compared to those who were not on diuretics (mean AAO = 63.3 ± 10.9 years; p = 0.000011). Those who started taking diuretics after onset of PD symptoms had a mean AAO similar to those never exposed (mean AAO = 65.5 ± 10.2 years; p = 0.23).

### Calcium channel blockers (CCBs)

131 patients were taking CCBs at time of first encounter. Of these, 46 were on CCBs prior to the onset of PD motor symptoms and 28 were started after. Details regarding when CCBs were started was unknown for 57 patients. The types of CCBs included amlodipine, diltiazem, nifedipine, and verapamil. PD patients who were on CCBs prior to onset of PD symptoms had later AAO (mean AAO = 71.5. ± 9.9 years) compared to those who were not on CCBs (mean AAO = 63.1 ± 10.8 years; p = 7.4 × 10^–7^). Those who started taking CCBs after onset of PD symptoms had an older mean AAO than those never exposed to CCBs (mean AAO 66.6 ± 10.8 years; p = 0.11).

### Anti-diabetic medications

122 patients were taking anti-diabetic medications at time of first encounter. 48 of these were on anti-diabetic medications prior to the onset of PD symptoms and 20 were started after. Details regarding the start of anti-diabetic medications could not be ascertained for 54 patients. Anti-diabetic medications included insulin, biguanides, thiazolidinediones, sulfonylureas, alpha-glucosidase inhibitors, SGLT2 inhibitors, DPP-4 inhibitors, and GLP-1 agonists. PD patients who were on anti-diabetic medications prior to onset of PD symptoms had later AAO (mean AAO = 68.5 ± 9.8 years) compared to those who were not (mean AAO = 63.3 ± 11.0 years; p = 0.0007). Those who started taking anti-diabetics after onset of PD symptoms also had a mean AAO older than those never exposed to these medications (mean AAO 68.1 ± 9.4 years; p = 0.034).

### Beta-agonists

56 patients were taking beta-agonists at time of first encounter. Of these, 25 patients were on beta-agonists prior to the onset of PD symptoms and 17 were started after. Details regarding when beta-agonists were started were unknown for 14 patients. A precise duration of treatment could not be ascertained with sufficient detail. PD patients who were on beta-agonists prior to symptom onset had later AAO (mean AAO = 68.8 ± 9.8 years) compared to those who were not on beta-agonists (mean AAO = 63.5 ± 10.9 years; p = 0.013). Those who started taking beta agonists after onset of PD symptoms also had a mean AAO older than those never exposed to beta-agonists, (mean AAO 68.3 ± 9.8 years, p = 0.11). Only 3 patients who were on beta-agonists had a smoking history within the past 10 years.

### Smoking history

Patients who were smokers within 10 years prior to first visit had a younger AAO (n = 56; mean AAO = 59.2 ± 8.2 years) compared to those who were not (n = 1085; mean AAO = 64.0 ± 10.8 years; p = 0.001; Fig. 1d).

### Family history

Patients with a family history of PD had a younger AAO (n = 310; mean AAO = 61.8 ± 10.2 years) compared to those who did not (n = 835; mean AAO = 64.4 ± 11.1 years; p = 0.0001).

### Multiple regression analysis

Multiple regression analysis was performed with data from 727 patients. 474 patients were not included as they had missing data values. This analysis identified AB (β = 5.7), statins (β = 5.6), and NSAIDs (β = 4.1) as the strongest independent predictors of AAO (p < 0.001). Smoking history (β = −6.0) was also a strong independent predictor (p = 0.001). ACE-I/ARBs, CCBs, diuretics, anti-diabetic medications, beta-agonists, and FH were not strong AAO predictors (Table 1).

## Discussion

Several medications, including statins, NSAIDs, anti-hypertensives, anti-diabetic medications, and beta-agonists were associated with older PD AAO in our cohort, while a smoking history and FH of PD were associated with younger PD AAO. A multiple regression model identified ABs, statins, and NSAIDs as strong independent predictors of older AAO of PD, suggesting that treatment with these medications may delay the onset of PD. On the other hand, smoking history was a strong independent predictor of younger AAO. 

The question whether statins may affect the risk of developing PD has received conflicting answers from multiple epidemiological studies. Similar to our findings, a retrospective study of 419 PD patients showed that those treated with cholesterol-lowering medications had a delayed disease AAO of 9 years (63.6 versus 54.6 years) when compared with PD patients who were not on lipid-lowering treatment, in addition to elements suggestive of slower disease progression [10]. Of note, in our larger PD cohort AAO was further delayed, in absolute terms, to an age of 70.8 years. Putative neuroprotective mechanisms attributed to statins include inhibition of proinflammatory substances, microglia, and oxidative stress, as well as suppression of alpha-synuclein aggregation [11]. Most, but not all, epidemiological studies found the use of statins to be associated with a reduced risk of developing PD [3]. However, a randomized clinical trial examining potential disease modifying effects of simvastatin failed to demonstrate any effect over 24 months in patients with moderate PD [12].

To our knowledge, this is the first study showing a delayed PD AAO in subjects exposed to AB, who developed PD symptoms almost a full decade later than those never exposed. However, when ABs were initiated after the onset of PD, AAO did not differ from the group never exposed, suggesting that the cardiovascular indication for AB use did not influence PD AAO. These results add to a large, albeit controversial, body of research on the effect of adrenergic modulating drugs on PD risk. The beta-2 adrenoreceptor is a regulator of the alpha-synuclein gene, and it has been hypothesized that beta-2 adrenoreceptor antagonists increase expression of alpha-synuclein, thus promoting the development of PD, while beta-2 adrenoreceptor agonists decrease this expression and are thus protective [8]. In accordance with this theory, a decreased risk of PD with salbutamol use and an increased risk of PD with propranolol use was reported from a large population database [8]. Several studies casted doubt on these data, noting that beta-2-agonists are typically used in smoking-related pulmonary conditions, likely associated with a reduced risk to develop PD, and beta-2-antagonists—in particular propranolol – are frequently prescribed for tremor. Indeed, propranolol was prescribed after the onset of motor symptoms in over 90% of cases in our PD cohort, under an incorrect initial diagnosis of essential tremor, supporting the suggestion that it may not be truly associated with increased PD risk when adjusting for clinical indication [4]. Interestingly, other adrenergic blockers not used to treat tremor (i.e. carvedilol and metoprolol) were associated with a lower risk of developing PD [4]. Independent studies demonstrated that carvedilol was associated with a reduced PD risk when prescribed over a year prior to diagnosis [13], while other investigations have yielded conflicting results [14]. Although they also appeared to have a 5-year delayed PD AAO, very few patients on beta agonists were recorded in our cohort, preventing a meaningful statistical analysis.

NSAIDs, and most notably aspirin, have been associated with older AAO of PD, with stronger association noted with a higher dose of aspirin or a longer aspirin intake duration [2]. NSAIDs have been associated with a reduced PD risk in some studies, although data from other studies is controversial [7]. Neuroinflammation has been implicated as a potential mechanism, supported by alterations of inflammatory markers in serum and cerebrospinal fluid of PD patients, as well as neurohistological and neuroimaging evidence of inflammation [15].

The possible reasons why a history of smoking would predict a younger PD AAO in our cohort appear less intuitive. Notably, the percentage of subjects who had been active smokers was quite small, less than 5% of the total dataset, which may have biased the results. This particularly low prevalence may be explained by rates of smoking, which are substantially lower in California (about 12.3% in 2008) [16] than in Europe (about 27.2% in 2010) [17]. While smoking is generally associated with a reduced PD risk [2], the relationship of smoking with PD AAO is more controversial. One review found ten studies that showed a delay in AAO, two studies that showed an earlier AAO, and nine studies without a directional effect [2]. We also did not have information regarding smoking-pack-years, which could yield more differentiated results. We postulate that these patients may have already had a strong predisposition to developing PD, thus overcoming the possible protective effect of smoking. One fourth of smokers had a positive family history for PD, but the remaining did not have an identifiable risk factor. Only one patient on beta-agonists was a smoker. Another consideration is that smoking may be more common among younger age groups, and thus we are capturing younger subjects that smoked recently rather than older subjects who smoked over 10 years ago.

Patients being treated for T2D in one study developed PD about 7 years later (66.9 years) than patients who did not have T2D (60.7 years) or patients who developed diabetes after PD onset (60.6 years) [9]. AAO for PD patients who had been on anti-diabetic treatment longer than 7 years had a greater delay than those treated for 7 years or less [9]. In our analysis, out of the 122 patients on anti-diabetic medications at first encounter, we were unable to ascertain start time for 54 patients and thus none of them were included in the regression model, which may have reduced statistical power. Given the potential disease modifying effect of glucagon-like peptide receptor agonists in PD patients [18], the role of anti-diabetics should be explored further.

The medications associated with a delayed AAO in our study appear to have a few common links that may explain a disease modifying effect. First, many of them are antihypertensives, which may modulate the effects of hypertension as a risk factor for PD [19]. In addition, all medications that appear to modulate PD risk in this study have a direct or indirect effect on the sympathetic nervous system and neuroinflammatory response, which may have a prominent role in the pathogenesis of PD [20]. Sympathetic hyperactivity has a major influence on neuroinflammatory status through the inflammasome NLRP3–IL-1β axis, either as a trigger or a consequence of increased cytokine release, and these inflammatory markers have been reported as critical signaling molecules of immune activation in the central nervous system [20]. In addition to suppressing sympathetic overactivity and reducing (and potentially reversing) cardiac remodeling [21], AB have anti-inflammatory effects, as demonstrated by their attenuation of cytokine storm-induced lung injury in COVID-19 [22]. Similarly, statins have been shown to decrease sympathetic activity [23], as well as enhance anti-inflammatory and inhibit pro-inflammatory functions within microglia in vitro [24]. NSAIDs inhibit prostaglandin synthesis, which has been described to decrease plasma norepinephrine and thus reduce sympathetic function [25]. In preclinical studies, carnosine, which acts by suppressing sympathetic activity [26], slowed progression of motor deficits and alpha-synuclein deposition in the Thy1-aSyn mouse [27]. The role of the noradrenergic system in PD deserves further evaluation in human studies [28].

A major limitation of this study lies in its retrospective nature. Information regarding specific medication timing was obtained by chart review or by patient report, both of which may be inaccurate for multiple reasons including recall bias or lack of data regarding compliance. Some data points had to be excluded from the multiple regression analysis because we were unable to establish medication timing relative to development of PD symptoms. In addition, we were unable to precisely determine exposure time and dose effect. Small sample sizes also limit the interpretation of the data. Finally, since caffeine intake was not included in our standardized template, we could not correct risk modifying data for coffee exposure, a behavior previously associated to delayed PD AOO [2].

A final consideration is whether age may be a confounding factor, as older patients – who are more likely to develop PD—are also more likely to be on a number of these medications. Large surveys reported a similar proportion of patients within 18–65 years of age on AB as compared to those above 65 years of age [29]. On the other hand, different proportions of patients appear to be treated with cholesterol medications depending on age [30].

In conclusion, our study demonstrated that common medications including AB, statins, and NSAIDs are associated with a delayed onset of PD. Possible mechanisms modulating the risk of neurodegeneration include hypertension control, down-regulation of the sympathetic nervous system and anti-inflammatory actions. Research using a prospective study design in large cohorts is needed to examine these effects, as drug repurposing would have important advantages to PD patients and the healthcare system.

## Supplementary Information

Below is the link to the electronic supplementary material.

Supplementary file1 (DOCX 378 KB)

## Data Availability

All the data and information associated with this manuscript are available upon reasonable request.
