# Supplementary material for: Adrenergic blockers, statins, and non-steroidal anti-inflammatory drugs are associated with later age at onset in Parkinson’s disease
Source: J Neurol. 2025 Mar 6;272(3):255. doi: 10.1007/s00415-025-12989-2 (PMC11885381; doi:10.1007/s00415-025-12989-2)

**Supplementary Materials:**

**Adrenergic blockers, statins, and non-steroidal anti-inflammatory drugs are associated with later age at onset in Parkinson’s disease**

Camille Malatt, MD, Helia Maghzi, MD, Elliot Hogg, MD, Echo Tan, MD, Ishani Khatiwala, MD, Michele Tagliati, MD

Department of Neurology, Cedars-Sinai Medical Center, Los Angeles, CA

**Corresponding author:**

Camille Malatt, MD

127 S. San Vicente Blvd. #A6600

Los Angeles, CA 90048

(310)-423-6472

[Camille.malatt@cshs.org](mailto:Camille.malatt@cshs.org)

**Table S1:** Multiple regression analysis using AAO as the dependent variable, with gender and the described risk-modulating factors as binary covariates. Formula used was lm(formula = AAO ~ ABs + ACE-Is/ARBs + CCBs + Diuretics + Anti-diabetic Medications + Statins + NSAIDs + Beta-agonists + Smoking history + FH + Gender, data = df)

| **Variable** | **Estimate** | **Standard error** | **T value** | **P value** |
| --- | --- | --- | --- | --- |
| (Intercept) | 61.9866 | 0.7112 | 87.155 | < 2e-16 |
| ABs | 5.7123 | 1.3972 | 4.088 | 4.84e-05 |
| ACE-Is/ARBs | 1.5926 | 1.2551 | 1.269 | 0.204868 |
| CCBs | 2.8434 | 1.7594 | 1.616 | 0.106518 |
| Diuretics | 0.8742 | 1.8911 | 0.462 | 0.644037 |
| Anti-diabetic Medications | -1.7958 | 1.8685 | -0.961 | 0.336834 |
| Statins | 5.6217 | 1.1100 | 5.065 | 5.21e-07 |
| NSAIDs | 4.0640 | 1.2078 | 3.365 | 0.000807 |
| Gender | -1.2042 | 0.7993 | -1.507 | 0.132366 |
| Beta-agonists | 0.7671 | 2.2382 | 0.343 | 0.731907 |
| Smoking history | -5.9562 | 1.8154 | -3.281 | 0.001085 |
| FH | -1.5582 | 0.8707 | -1.790 | 0.073950 |

**Fig. S2:** Dot plots showing the association of PD AAO with a) ACE-I/ARB intake, b) beta-agonist intake, c) CCB intake, d) diuretic intake, e) anti-diabetic medication intake, f) family history, and g) gender. Black bar is the mean AAO and dotted bars are +/- 1 SD.


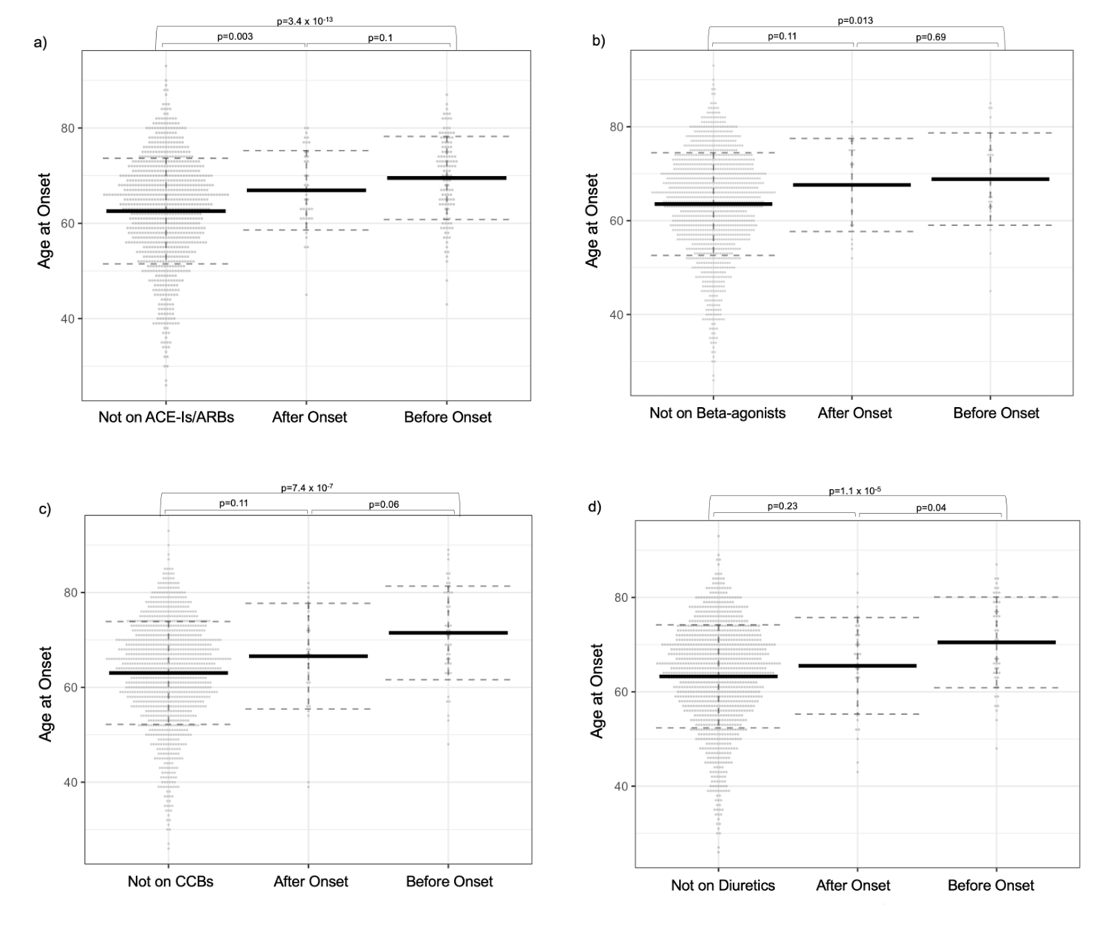

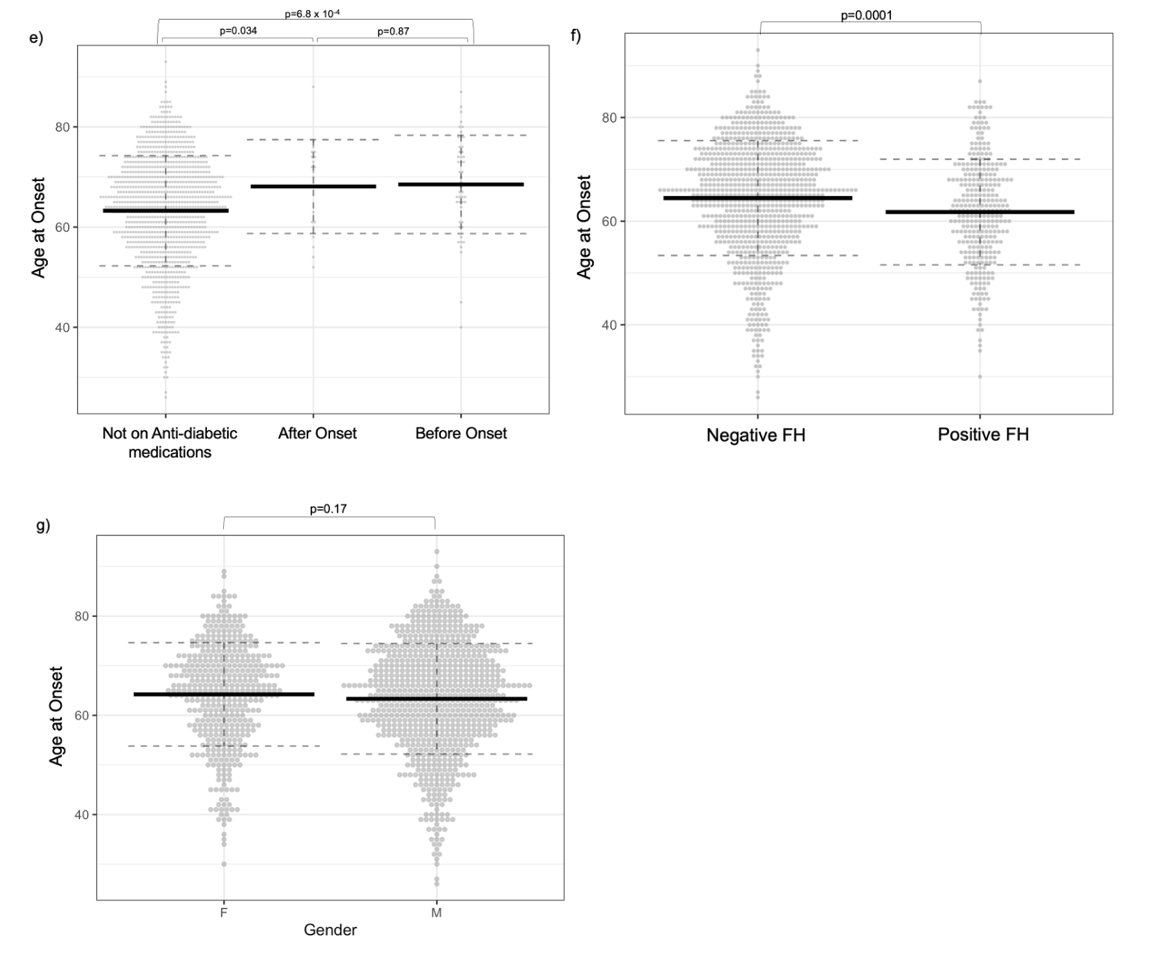

Supplement: Supplementary file 1 — Supplementary file1 (DOCX 378 KB) [file 415_2025_12989_MOESM1_ESM.docx]
